# The Patient Reported Outcomes, Burdens and Experiences (PROBE) Project: development and evaluation of a questionnaire assessing patient reported outcomes in people with haemophilia

**DOI:** 10.1186/s40814-018-0253-0

**Published:** 2018-02-27

**Authors:** MW. Skinner, C. Chai-Adisaksopha, R. Curtis, N. Frick, M. Nichol, D. Noone, B. O’Mahony, D. Page, J. S. Stonebraker, A. Iorio

**Affiliations:** 1 Institute for Policy Advancement Ltd, 1155 23rd Street NW #3A, Washington, DC 20037 USA; 2 Department of Medicine, McMaster University, Hamilton, Canada; 3 Factor VIII Computing, Berkeley, USA; 4 National Hemophilia Foundation, New York, USA; 5 Sol Price School of Public Policy, University of Southern California, Los Angeles, USA; 6 Irish Haemophilia Society, Dublin, Ireland; 7 Trinity College Dublin, Dublin, Ireland; 8 Canadian Hemophilia Society, Montreal, Canada; 9 Poole College of Management, North Carolina State University, Raleigh, USA; 10 Department of Health Research Methods, Evidence, and Impact, McMaster University, Hamilton, Canada

**Keywords:** Haemophilia, Patient-reported outcomes, Quality of life, Patient-centered research

## Abstract

**Background:**

The interest of health care agencies, private payers and policy makers for patient-reported outcomes (PRO) is continuously increasing. There is a substantial need to improve capacity to collect and interpret relevant PRO data to support implementation of patient-centered research and optimal care in haemophilia. The Patient Reported Outcomes, Burdens and Experiences (PROBE) Project aims to develop a patient-led research network, to develop a standardized questionnaire to gather patient-reported outcomes and to perform a feasibility study of implementing the PROBE questionnaire.

**Methods:**

A pilot questionnaire was developed using focus group methodology. Content and face validity were assessed by a pool of persons living with haemophilia (PWH) and content experts through interactive workshops. The PROBE questionnaire was translated with the forward-backward approach. PROBE recruited national haemophilia patient non-governmental organizations (NGOs) to administer the questionnaire to people with and without haemophilia. PROBE measured the time to complete the questionnaire and gathered feedback on its content and clarity; staff time and cost required to implement the questionnaire were also collected.

**Results:**

The PROBE questionnaire is comprised of four major sections (demographic data, general health problems, haemophilia-related health problems and health-related quality of life using EQ-5D-5L and EQ-VAS). Seventeen NGOs participated in the pilot study of the PROBE Project, recruiting 656 participants. Of these, 71% completed the questionnaire within 15 min, and all participants completed within 30 min. The median total staff and volunteer time required for the NGOs to carry out the study within their country was 9 h (range 2 to 40 h). NGO costs ranged from $22.00 to $543.00 USD per country, with printing and postage being the most commonly reported expenditures.

**Conclusions:**

The PROBE questionnaire assesses patient-important reported outcomes in PWH and control participants, with a demonstrated short completion time. PROBE proved the feasibility to engage diverse patient communities in the structured generation of real-world outcome research at all stages.

**Trial registration:**

Trial registration: NCT02439710.

**Electronic supplementary material:**

The online version of this article (10.1186/s40814-018-0253-0) contains supplementary material, which is available to authorized users.

## Background

Haemophilia is an inherited bleeding disorder, caused by a deficiency of coagulation factor VIII (haemophilia A) or factor IX (haemophilia B). Persons living with haemophilia (PWH) experience various degrees of bleeding, depending on residual coagulation factor levels [1]. Bleeding occurs most commonly in joints, soft tissue and muscles [2], causing short-term symptoms (acute bleeding, acute pain) [3] and long-term complications (chronic pain, haemophilic arthropathy, or disability) [4, 5]. Acute and chronic complications result in a major impact on health-related quality of life (HRQoL) of PWH.

The mainstay of treatment in PWH is factor replacement therapy, for either prevention of bleeding (prophylaxis) or treatment of bleeding on an on-demand basis [6]. Prophylaxis has been shown capable of maintaining joint status and function [6], but adequate prophylaxis requires lifetime self-administration of factor concentrate two to three times per week. Moreover, PWH who suffer from haemophilic arthropathy require a multimodal approach, including medical treatment, surgery, rehabilitation and exercise [7–9]. Assessment of patient-important outcomes beyond bleeding frequency and functional status, including burden of treatment, impact on lifestyle and life choices, is a major aspect that needs to be quantified. Recently, the National Hemophilia Foundation, McMaster University *Guideline on Care Models for Haemophilia Management*, identified the important outcomes that patients value [10]. Those outcomes are mortality, missed days of school or work, number of emergency room visits, length of hospital stay, quality of life, joint status, educational attainment, patient adherence and patient knowledge. However, bleeding or bleeding rates were not considered as an important outcome.

Research including or principally focusing on real-world evidence and patient-reported outcomes (PRO) is increasingly valued by relevant stakeholders, including governments, regulators [11–13], health care agencies, health technology assessment (HTA) agencies [14], private payers and policy makers [15, 16]. Patients have unique perspectives and may consider issues differently than regulators, manufacturers, scientists, clinicians and payers [11]. The ability to collect and interpret relevant PRO to support the implementation of the prophylactic treatment, home care and integrated disease management (“comprehensive care”) is becoming more and more relevant to ensure optimal care to PWH [12, 15]. Over the last few years, direct patient involvement in designing, conducting and evaluating research has emerged as a theme in Europe and North America to ensure research is centered on patients and their needs [13].

The Patient Reported Outcomes, Burdens and Experiences (PROBE) Project is a research initiative conducted by PWH for PWH, with the specific aim to contribute PRO relevant to haemophilia by direct patient involvement in the design, conduct, analysis and reporting of patient-centered outcome research in the field of haemophilia.

## Methods

### General objectives

**Fig. 1:**
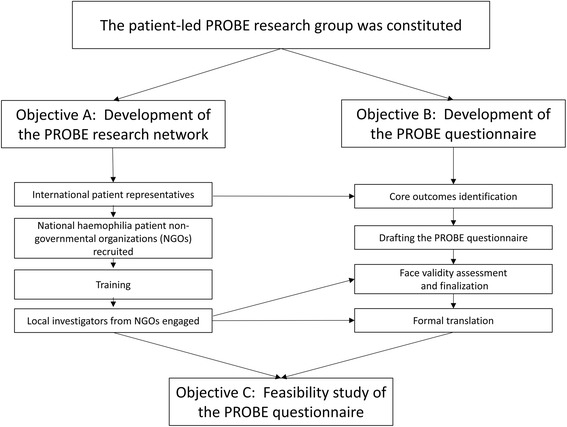
Flow diagram of the PROBE study

The PROBE Project aims to implement a structured data collection of PRO across several countries and cover a wide spectrum of health economic environments, to build a robust evidence base for comparative effectiveness, outcome research, evidence-based decision making and advocacy. To accomplish these overarching goals, three intermediate objectives have been identified for this phase of research: (a) develop a patient-led research network, (b) develop a standardized questionnaire to gather PRO and (c) perform a feasibility study. Figure 1 demonstrates the flow diagram of the process of the PROBE Project.

### The research group

Upon a call to action [16] of the PROBE principal investigator (MS), a group of patients (RC, BOM, DN, DP, MS), supported by an epidemiologist (AI), an expert in outcome research (MN), an expert in management science and economic engineering systems (JS) and representatives of patient organizations (NF, BOM, DP), convened as co-investigators to form the PROBE research group (investigators) of the Patient Outcomes Research Group Ltd. (PORG) (Washington, DC, USA), the enterprise founded to administer the independent investigator-initiated research and grant funds the group has secured (see the “Acknowledgements” section). CC, a PhD candidate in Health Research Methodology at McMaster University, has worked closely with the investigators to support the technical aspects of the project development and data analysis. PORG has entered a research agreement with the Health Information Research Unit at McMaster University, Hamilton, ON, Canada, where the PROBE research database was established.

Co-investigators RC, NF, AI, DN, DP, JS, MN and BOM joined the group subsequently. PORG was incorporated in Washington, DC, USA, on 6 June 2016.

### The development of a patient-led research network

The investigators identified and invited a selected group of national haemophilia patient non-governmental organizations (NGOs) to join the PROBE Project forming the PROBE research network. The proposed return for the investment were discussed with each NGO representative before developing a structured research protocol (NCT02439710). The selection of the countries for the pilot was performed by the investigators based on the following parameters: regions (North America, South America, Europe, Western Pacific, Asia or Africa), diversity in access to treatment product, health care system development level, health benefit coverage model and payer typology and local capacity to implement the requested operations. Each participating NGO designated a local investigator who managed the recruitment of study participants and executed the feasibility assessment of PROBE in their country.

### The development of the PROBE questionnaire

The PROBE questionnaire was developed, refined and finalized by the PROBE research group, with input at several stages from local investigators and patient representatives belonging to NGOs within the PROBE research network. The PROBE questionnaire was developed with the dual objective of enabling fast and efficient collection of relevant information for advocacy purposes directly from patients and generating broad awareness of the importance of research led by patients for patients. It was explicitly discussed within the research network that additional scientific aspects of the PROBE questionnaire development and its characteristics would be pursued during later stages. As well, it was clearly stated that the prototypal version of the PROBE questionnaire was not expected to be the only or even main questionnaire developed by the research group.

The questionnaire was *developed* beginning with a brainstorming exercise of 48 participants from 18 countries. After being primed with a presentation reviewing existing tools measuring PRO, the participants were tasked with identifying and ranking key PRO and issues related to their measurement and evaluation. The PROBE research group summarized the results of the brainstorming exercise and produced a first draft of a questionnaire. The questionnaire was *refined* using multiple focus groups of PWH and people not personally affected by haemophilia. A full psychometric analysis including a validation study and test-retest reliability study was scheduled upon the successful completion of this refinement phase. They were performed and are reported elsewhere (manuscript submitted). Once all input from the focus groups were incorporated, the questionnaire was *finalized* by assessment for readability using the Flesch-Kincaid Grade Level classification to judge reading level [available at https://readable.io/], edited accordingly and translated into all major languages spoken in the countries participating in the PROBE research network.

### The feasibility study

The usability and properties of the finalized PROBE questionnaire were tested in a study involving individuals recruited by the NGOs.

#### Study population and enrollment

Participants were recruited through NGOs utilizing their existing membership rosters, social media outlets and meetings or events. Participants were required to meet one of two simple inclusion criteria: (1) PWH (haemophilia A or B with any degree of disease severity) or (2) “Controls” (individuals with no bleeding disorders). Except where required by local regulation, no pre-defined age limits were imposed on study participation. Investigators planned a post hoc analysis to assess a minimum age or cut off standard for PROBE data analysis. Based on the analysis, investigators agreed to include participants who were greater than age 10, a point at which responses were no longer interpretable or logically consistent with anticipated responses. Additionally, PWH typically begin to learn self-management and self-infusion skills in early adolescence (10–12.5 years) [17] which further supports adoption of the age limit selected by the investigators. Parents and caregivers of PWH were instructed to take the survey for themselves (as Controls) and not to answer for their child. Recruitment of approximately 50 participants (as a sum of PWH and Controls) per each of the participating countries, aiming at a minimum of 600 filled questionnaires across all countries, was planned.

#### Ethical considerations and privacy

The academic partner hosting the PROBE Project database (McMaster University) obtained Review Ethical Board approval and Privacy Officer clearance for PROBE from the Hamilton Integrated Research Ethics Board. Participating NGOs obtained or did not seek ethical approval depending on the local regulation for questionnaire-based studies led by patients. Customary processes were implemented to protect individual patient identities. Specifically, no patient identifier or personal information was collected as part of the study. Upon completion, questionnaires were immediately collected by local country investigators, bundled and shipped directly to the data coordinating center at McMaster University for data entry. Even though data were collected at the individual PWH and Control level, reports were limited to aggregate data to perform comparison of outcomes at the population level.

#### Outcome measures

The principal objective of the study was to assess feasibility and impact of implementing the PROBE questionnaire by the participating NGO, measured by staff and volunteer time required, cost incurred and barriers encountered. Free text comments and questions on the questionnaire were invited from participating NGOs via a separate questionnaire (Additional file 1).

#### Data analysis

Questionnaires were filled on paper and shipped to the data coordinating center at McMaster University for input into the PROBE electronic database. Descriptive statistical analysis was used as appropriate. Response rate was measured as a proportion of respondents returning the survey. Time to completion for each participant was collected as an ancillary question with the study questionnaire, and cost per completed survey was calculated by dividing the total cost (converted to USD) reported by the NGOs with the number of collected surveys.

## Results

### Development of the patient-led research network

We successfully engaged national haemophilia organizations from 18 countries to participate in the PROBE Project. Patients from those national haemophilia organizations joined the process of questionnaire development. Over 500 participants were enrolled for the feasibility study through the PROBE research network.

### Development, refinement and finalization of the PROBE questionnaire

#### Development

Early papers that pioneered the concept of collecting comparative outcome data across countries within the haemophilia community informed the concept development for PROBE [14, 18]. Forty-eight delegates from 18 countries and a range of backgrounds within the haemophilia community (PWH, parents, caregivers, haemophilia specialists and researchers) gathered at the Haemophilia Experiences, Results and Opportunities Summit (HERO Summit). A workshop within the HERO Summit was dedicated to identifying and agreeing on key issues related to data capture, outcome measurement and assessment. Over 2 days, delegates discussed the lack of patient-reported outcome data and widely used PRO assessment tools to measure the full range of effects of comprehensive/expert care, home treatment and specific treatment regimens; identified opportunities and issues in developing a patient research framework for establishing appropriate metrics; listed and ranked outcomes important to PWH, health care professionals, governments and payers along with covariates to consider, surrogates and alternative data elements; and designed a framework to pilot a research project. Critical outcomes and relevant metrics of importance to the haemophilia community were identified (including quality of life, family burden, education/school, employment, treatment approaches, therapeutic outcomes and activities of daily living). These outcomes and related identified metrics provided the basis for the draft version of the PROBE questionnaire and feasibility study. The final core set of outcomes was ultimately determined through group discussion of all delegates. Table 1 summarizes the identified outcomes of importance and metrics to consider.

**Table 1:** Summary of the outcomes of importance and metrics to consider

Outcomes of importance	Relevant metrics to consider
Reduced burden of living with haemophilia	
• Life • Family • Education/school • Employment • Activities	• Family life, marital status, children • Educational attendance, attainment • Employment duration, underemployment, attendance • Impact on daily living, activities of daily living, mobility impairment, assistance required • Current health status (HRQoL)
Reduced complications associated with haemophilia and treatment	
• Joint disease • Pain/depression/anxiety • HIV/HCV • Obesity • Other comorbidities	• Joint status • Pain (chronic, acute, interference with activity, timing, medication) • Depression • Resource utilization • Mortality, longevity

#### Refinement

Focus groups of PWH were used for testing the draft version of the PROBE questionnaire. Three parallel focus groups were organized in conjunction with a planned Global Advocacy Leadership Summit (Global Leadership Summit) representing 18 countries (Argentina, Australia, Brazil, Canada, Colombia, France, Germany, Hungary, Ireland, Italy, Japan, Mexico, The Netherlands, New Zealand, Spain, the UK, the USA and Venezuela). Thirty-seven subjects participated in the focus groups including 20 PWH and 17 participants without a bleeding disorder (e.g. haemophilia carriers, haemophilia specialists, nurse practitioners and physiotherapists). The focus group participants reviewed the introductory description of the PROBE project, stated objectives and provided further insights on the draft questionnaire content, clarity and relevance. After the focus groups, additional one-on-one interviews were conducted with some focus group participants to further clarify the focus group input. Interviews were continued until there were no more questions about the meaning of questionnaire items. In brief, patient focus groups were instrumental in helping to shape the questionnaire definitions of acute and chronic pain and identify items for pick lists of potential responses, e.g. areas of daily living where they had difficulty, and relevant comorbidities, e.g. oral health-related and gynecological issues. Questions were reworded using both patient-friendly terminology or split in two versions to compare clinical and patient-friendly definitions, e.g. for target joints. In several instances, options for the answers were expanded to cover the need of different countries, e.g., when asking about assistive devices in use. The contribution of the focus group in terms of wordsmithing, editing and clarification process was key to improve clarity, crispness and face validity for living experiences of the patients.

#### Finalization

The questionnaire was reassessed by the investigators for completeness of the questionnaire for assessing all important outcomes, clarity and face validity. Investigators sought additional feedback by inviting comment on the redrafted questionnaire from patient representatives from some of the countries that participated in the Global Leadership Summit, and not otherwise represented by the home countries of the investigators. The US English version of the questionnaire was reviewed and assessed for readability using the Flesch-Kincaid Grade Level classification to judge the reading level [available at https://readable.io/]. After editing, the text received a Flesch-Kincaid grade level of 7.6. The PROBE questionnaire was then translated and localized for 18 countries using an 8th grade language standard. Twenty local versions were produced: English (Australia, Canada, England, Ireland, New Zealand, USA), French (Canada, France), German (Germany), Hungarian (Hungary), Italian (Italy), Japanese (Japan), Dutch (The Netherlands), Portuguese (Brazil) and Spanish (Argentina, Colombia, Mexico, Spain, the USA and Venezuela). Language-specific versions were produced using the standard forward-backward technique and checked for appropriateness by at least one mother tongue field expert. The professional translation agency CETRA Language Solutions (Elkins Park, PA, USA) was contracted for the translation and grade level scoring. Country-specific language versions of the EuroQol 5-dimension 5-level instrument (EQ-5D-5L), a standardized measure of health status developed by the EuroQol group, consists of the following dimensions, mobility, self-care, usual activities, pain or discomfort and anxiety or depression, and the EuroQol visual analog scale (EQ-VAS) were incorporated and used with permission of the EuroQol Research Foundation [19]. The EQ-5D-5L and EQ-VAS were incorporated in the PROBE questionnaire in a format equivalent to their source versions without alteration.

### The PROBE questionnaire

The PROBE questionnaire utilized in the feasibility assessment had a total of 29 questions (note: Based upon lessons learned from this feasibility assessment and subsequent phases of research, the PROBE questionnaire has been minimally updated and refined for use in future phases of research). For this research phase, *Section I* asks for demographic data (country, gender, body weight, age, education and marital and family status) and *Section III* asks for “haemophilia-related questions” (disease severity, inhibitor status and history, bleeding frequency and history, treatment regimen and history and presence of target joint(s) and range of motion limitations). These two sections (Section I and Section III) do not cover any PRO domains but provide information needed to explore and interpret the properties of the PRO sections. Should the PROBE survey be run as part of a clinical study or haemophilia registry, these parts could be omitted if otherwise covered and available for analysis. The other sections of the questionnaire cover the following domains: pain, independence, activities of daily living, educational attainment, employment and relationships. *Section II*, “general health problems”, includes the use of mobility aids and assistive devices, use of pain medication, presence of and how acute pain and chronic pain interfere with daily life, difficulty with daily activities, surgical history and concurrent medical problems and work/school life. Scaling responses are dichotomous (yes or no) and frequency. Frequency questions are answered on a 7-point Likert scale (never, rarely, occasionally, sometimes, frequently, very frequently and all of the time). *Section IV* includes the EQ-5D-5L and the EQ-VAS of global health [19].

### The feasibility study

**Fig. 2:**
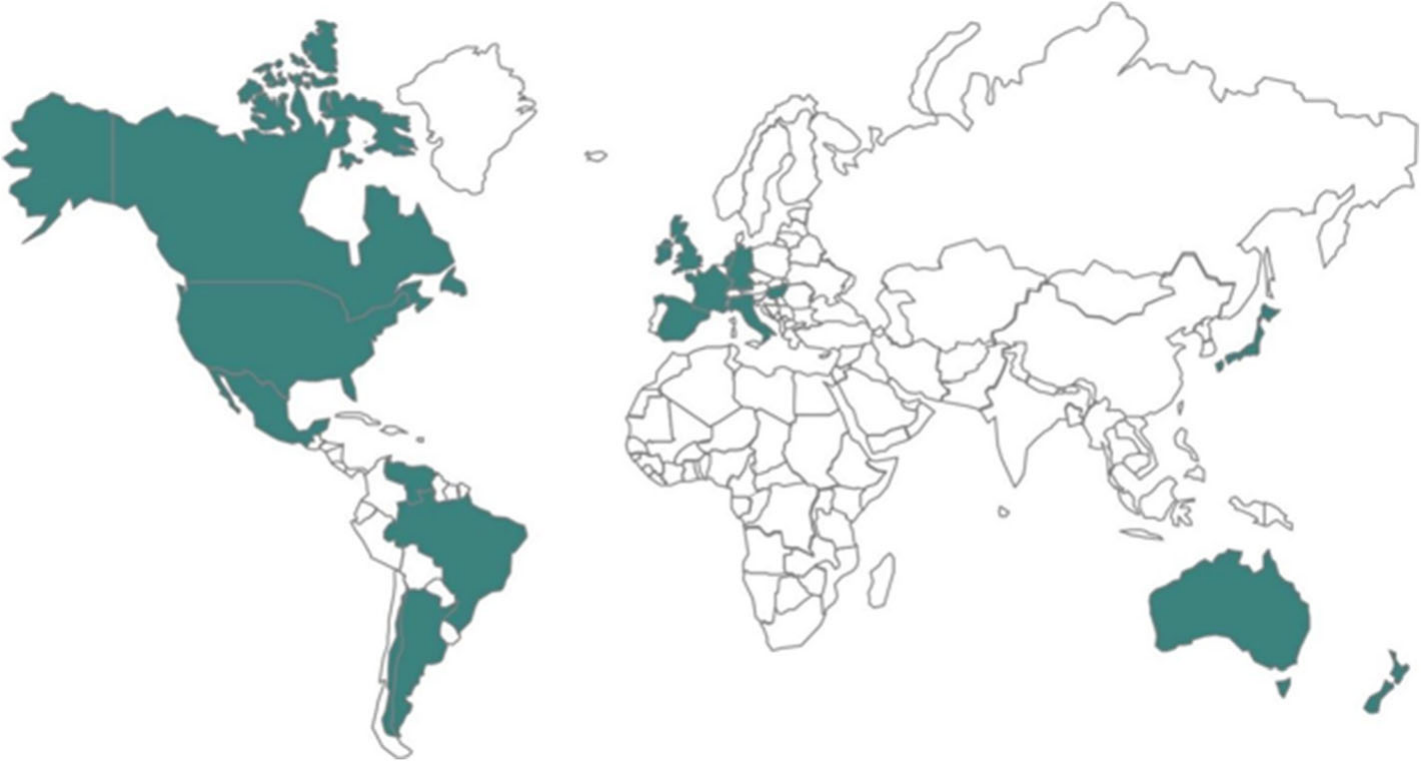
Participating countries: Argentina (Cordoba Chapter), Australia, Brazil, Canada, France, Germany, Hungary, Ireland, Italy, Japan, Mexico, The Netherlands, New Zealand, Spain, the UK, the USA, Venezuela

**Fig. 3:**
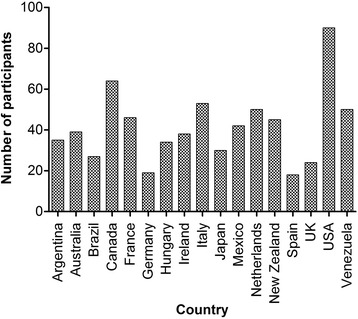
Number of participants by country

Patient organizations from 17 of the 18 mid to highly developed countries that attended the Barcelona Summit enrolled to participate in this study. Figure 2 depicts country participation. Each NGO distributed the PROBE questionnaire either by post, e-mail, at meetings, in-person or using other methods (clinic, camp or webpage). Figure 3 depicts the number of participants by country. Participants filled the questionnaire from April 2015 to December 2015. A total of 656 responses were collected and included in the analysis. Mean age was 41.9 years (SD 17.0). Of these, 353 were PWH with type A, 78 PWH with type B, 212 participants had no bleeding disorder (control population) and 13 were unknown as to bleeding disorder status.

**Fig. 4:**
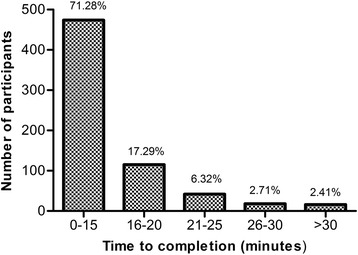
Time to completion of the PROBE questionnaire

The median response rate by country was 35.5% (Q1, Q3—14.0%, 78.0%). Fifty percent of the responders filled out the questionnaire on the same day they received it. The rest of participants returned the questionnaire 1 to 42 days after. Time to completion is shown in Fig. 4. Most participants (474, 71.28%) completed the questionnaire within 15 min, and all participants completed it within 30 min. Table 2 shows the time devoted by staff and volunteers to implement the study by country. Forty percent of NGOs rated the number of hours used to run the survey as “minimal”, 13% as “moderate”, 20% as “acceptable” and 3% as “significant” (23% did not respond for this item). Table 3 summarized the major themes of the barriers identified by the NGOs in implementing the study reported.

**Table 2:** Staff time and cost to implement the PROBE questionnaire by country

Country	Number of hours of paid staff used in performing the survey (h)	Number of hours of volunteer staff used in performing the survey (h)	Total time
Argentina	0	7	7
Australia	10	4	14
Brazil	6	15	21
Canada	2	0	2
England (UK)	3	20	23
France	4	8	12
Germany	5	4	9
Hungary	0	6	6
Ireland	2.5	0	2.5
Italy	0	24	24
Japan	0	8	8
Mexico	1	3	4
Netherlands	18	14	32
New Zealand	30	10	40
Spain	NR	NR	NR
USA	5.25	0	5.25
Venezuela	NR	NR	NR

*NR* not reported

**Table 3:** List of barriers of carrying out the PROBE study

Theme	Barriers
Instructions	Some parents or caregivers did not understand they were being asked to also complete the survey for themselves to serve as part of the control population. Similarly, some participants were not clear if their parents or caregivers could help them to fill out the questionnaire. Further clarification in the instructions was suggested.
Dissemination method	When the study was conducted as part of a meeting, and participants were asked to return surveys to a booth after completion, most never returned them despite labels requesting them to do so. Likewise, it was noted that handing it out in a meeting and asking people to return by mail is not an effective strategy as the return rate is low. It was recommended to have a dedicated time allotted to completing the survey when being conducted within an NGO meeting rather than distributing and collecting later.
Electronic completion	PDF format of the survey not designed to complete on a computer. Respondents required to print to complete surveys disseminated electronically. An on-line version of the survey was suggested. Also, low response for surveys disseminated via social media or email possibly due to summer holiday.
Time required	Conference participants were reluctant to complete what they perceived to be a time-consuming survey, when they had already completed several evaluation forms at the same meeting. Recommendation to NGOs to manage the total number of surveys conducted in one meeting. Despite the overall time to completion of ~ 15 min, a couple of people still found it long and/or daunting.
Ethics approval	Human research ethics committee took a long time for the approval (one country only). Longer lead time to prepare for implementation of the survey is needed if ethics approval will be required.

*PROBE* The Patient Reported Outcomes, Burdens and Experiences, *NGO* non-government organization

## Discussion

PROBE Project study results demonstrated the feasibility of planning and implementing a patient-led research network to explore PRO. The PROBE research group developed and tested a survey instrument aimed at collecting PROs in PWH and Controls. A network of NGOs in 17 countries, who collected 656 surveys using 20 localized language versions, was established. Results have demonstrated face validity, ease of administration and a short compilation time with minimal impact on NGO human and economic resources.

The true element of novelty of the PROBE Project is the leading role of the patients. There is an increasing awareness of the value of patient involvement in research [20]. The European Organization for Rare Diseases (EURORDIS) conducted a survey of 772 patient organizations [21]. This study found that non-financial support of patient organizations included facilitation of research projects and clinical trials by highlighting patients’ needs and expectations [21]. In the field of haemophilia, the PROBE Project is one of the few to outline the value and potential of direct patient involvement in planning and conducting research. The results of this study demonstrate the potential for an enhanced role of haemophilia patient organizations’ ability to implement research studies, collect patient-reported outcomes in a real-world setting and contribute credible data suitable for use in evidence-based decisions.

A second element of novelty is the PROBE questionnaire. The main goal in developing the PROBE questionnaire was to enable the collection of data to be used to improve treatment for haemophilia, including supporting comparative effectiveness analysis. For those health care settings moving toward value-based health care [22, 23], the capacity to measure and compare the impact of disease and treatment interventions on the life experiences of patients is critical. In this perspective, it is essential to measure HRQoL with a non-specific instrument, to allow comparison with medical interventions in other disease fields. The systematic review of the literature confirmed the previous finding that EQ-5D was one of the generic tools used most frequently in studies investigating HRQoL in PWH [24]. For this reason, the EQ-5D-5L was included as part of the PROBE questionnaire. To support comparison of alternative treatment modalities and intensities, a set of haemophilia-specific questions, which will be tested for their discrimination capacity and responsiveness in subsequent phases of the PROBE development, was included. The face and content validity, performed as part of this study, revealed that the items of PROBE questionnaire were appropriate for assessing the PRO of interest.

A third novel approach of the PROBE Project was to administer the PROBE questionnaire to both PWHs and participants without bleeding disorders (Controls). For Controls, the questionnaire surveyed all the questions in Sections II and III, whereas the haemophilia-related health questions were omitted for participants without a bleeding disorder. Generating a large sample of benchmark measures in the non-haemophilia population across many countries will be critical in assessing the impact of haemophilia on PRO beyond the baseline impact that age, comorbidities and life events have on all people. To the investigators’ knowledge, few other PRO tools [25–27] have developed a similar approach.

The PROBE questionnaire was designed to be concise. Seventy-one percent of the participants completed the questionnaire within 15 min. Previous studies have shown that a shorter form of questionnaires was preferred by the participants [28, 29]. As a comparison, the Hemofilia-QoL tool is comprised of 68 items in 8 domains (physical health, physical role, joint damage, pain, treatment satisfaction, emotional functioning, mental health and social support) and is designed for adult haemophilia patients and cannot be used to compare with participants without haemophilia [27]. The interest of health care agencies, private payers and policy makers for patient-reported outcomes (PRO) is continuously increasing. Cost and expertise to develop a robust and reliable data collection and analysis plan is a known challenge for NGOs. There is a substantial need to improve capacity to collect and interpret relevant PRO data to support implementation of patient-centered research and advocacy to obtain optimal care in haemophilia. PROBE has demonstrated that it can reliably address this need and its related challenges through establishment of a low-cost, low-burden and reliable data collection framework.

There are some limitations in the PROBE study design. The response rate in the pilot study has been inferior to that in other formal questionnaire development processes, including in the field of haemophilia [30]. However, the response rate obtained should be considered against the pragmatic design of the feasibility study. Given the extensive iterative process of survey development, investigators assessed that larger sample sizes were not required to demonstrate feasibility of administration by NGOs, to assess time to completion or further demonstrate content and clarity which were the primary outcome measures for this phase of research. The questionnaire has been voluntarily administered to patients by each participating patient organization, without dedicated research personnel. Each NGO decided how to distribute the questionnaire to the participants, which certainly impacted the response rate, but also informed the investigators on future best practice to guide NGOs participating in subsequent phases.

PROBE was not specifically designed to investigate PRO in children. As noted in the study population description above, the PROBE study inclusion criteria and participant selection process allowed participants greater than age 10 to participate. Investigators will use these responses, jointly with the experiences gained with age-specific tools in subsequent analysis.

Finally, there are several factors that may impact the cost of questionnaire implementation in multiple countries which we have not been able to account for completely, for example, wage rate, cost of transportation rate, cost of mailing rate or number of questionnaire distributed and collected. Cost (in USD) to implement the survey varied from $22 to $543 (for a median of $8.50 per participant, range $1.20 to $64.30) with printing and postage (to mail out the survey or ship completed surveys back to the investigation team) being the most commonly reported expenditures. Those reporting higher cost included costs for NGO staff (local country investigators) to travel to a meeting location where the PROBE questionnaire was to be administered. The cost per questionnaire provided should be considered as a guide, not a final estimate.

## Conclusions

PROBE has developed a concise questionnaire for assessing PRO in PWH and people without a bleeding disorder. The PROBE questionnaire combines generic and disease-specific outcomes in 29 items, requiring 15 min for completion. PROBE proved the feasibility to engage diverse patient communities in the structured generation of real-world outcome research at all stages. Future studies will explore the measurement properties of the PROBE questionnaire in the research network.

## Additional file

Additional file 1: Participating NGO evaluation report. (DOCX 24 kb)

## Abbreviations

EQ VAS: EuroQol visual analog scale
EQ-5D-5L: EuroQol 5-dimension 5-level
EURORDIS: European Organization for Rare Diseases
HERO: Haemophilia Experiences, Results and Opportunities Summit
HRQoL: Health-related quality of life
HTA: Health technology assessment
NGO: Non-governmental organization
PRO: Patient-reported outcome
PROBE: Patient Reported Outcomes, Burdens and Experiences
PWH: Person living with haemophilia
