# Supplementary material for: The Patient Reported Outcomes, Burdens and Experiences (PROBE) Project: development and evaluation of a questionnaire assessing patient reported outcomes in people with haemophilia
Source: Pilot Feasibility Stud. 2018 Feb 27;4:58. doi: 10.1186/s40814-018-0253-0 (PMC5828307; doi:10.1186/s40814-018-0253-0)
Supplement: Supplementary file 1 — Participating NGO evaluation report. (DOCX 24 kb) [file 40814_2018_253_MOESM1_ESM.docx]

**Appendix 1** Participating NGO Evaluation Report

**Patient Reported Outcomes Burdens and Experiences Study**

**Organization Phase 1 Evaluation Report**

**1. Country:** _________________________

**2. Name of the Organization:** ___________________________________

**3. Primary Contact Details:**

Name: __________________________________

Phone Number: __________________________________

Email: __________________________________

| **4. What is the total number of members in your organization?** | No.: _________ | - Unknown |
| --- | --- | --- |
| **How many people with haemophilia does this include?** | No.: _________ | - Unknown |

**5. What methods were used to distribute the survey? Please check all that apply**

- In-person
- Organization meeting
- By Post
- By Email
- By Phone
- Other, please describe: __________________________________________________

**6. Were the participants notified in advance they would be surveyed?**

- Yes
- No

**7. Please fill in as much detail as possible on how the surveys were distributed.**

| **Method the survey was distributed?** | **Number of surveys distributed** | **Date surveys were sent out** | **Date of deadline for return of surveys** | **Number of completed surveys returned** | **Date last survey received** |
| --- | --- | --- | --- | --- | --- |
| ___________________ | No.: _______ | Date: _________ | Date: _________ | No.: _______ | Date: _________ |
| ___________________ | No.: _______ | Date: _________ | Date: _________ | No.: _______ | Date: _________ |
| ___________________ | No.: _______ | Date: _________ | Date: _________ | No.: _______ | Date: _________ |

**8. Number of hours of paid staff used in performing the survey:** ___________________

Approximate Total: _________ Hours

- None

**Is the number of hours by paid staff used to carry out the survey?**

- Minimal
- Moderate
- Acceptable
- Significant
- Excessive

**Would the time required by paid staff prevent you from carrying out the survey again?**

- Yes
- No

**9. Number of hours of volunteer staff used in performing the survey**: ___________________

Approximate Total: _________ Hours

- None

**Is the number of hours by volunteer staff used to carry out the survey?**

- Minimal
- Moderate
- Acceptable
- Significant
- Excessive

**Would the time required by Volunteer staff prevent you from carrying out survey again?**

- Yes
- No

**10. Were there any costs you incurred (e.g. printing, postage) when carrying out the survey?**

- Yes Total Amount (in local Currency): _____________
- No

**If yes, select all that apply**

- Stationary
- Sending Postage for Survey
- Return Postage for Survey
- Printing
- Travel
- Room hire / rental at organization meeting
- Return Postage to PROBE
- Other, Please Describe: ______________________________________________________

**Q 11. Were there any other barriers or issues that you faced when you were carrying out this survey?**

_______________________________________________________________________________________________________________________________________________________________________________________________________________________________________________________________

**Q 12. Are there are any areas of interest that you would have liked to have seen or expected to see in the survey?**

_______________________________________________________________________________________________________________________________________________________________________________________________________________________________________________________________

**Thank you in advance for your help on this project of global importance**
